# A Review of Interest to Health Workers

**Published:** 1920-07-03

**Authors:** 


### THE MALARIAL SURVEY IN ENGLAND.

### A Review of Interest to Health Workers.

Two circumstances prompt us to make special note of this contribution to public health matters in England, the basis of which is recounted in the new book on malaria by Col. S. P. James as reviewed elsewhere (p. 358). The first is that whatever the forecasts of last year may have been with regard to malaria in this country, the fact remains that to-day all central laboratories are meeting a very large increase of " positives " in the samples of blood sent to them for malarial examination. The second is that malaria as a disease has been considered to belong so essentially to the realms of tropical medicine that it is doubtful whether the average health worker in this country fully realises either the frequency with which he may come across the various agencies concerned in its spread or even actual instances of early infection in men, women, or children.

### The Meaning of a Survey.

In summary the main features of this survey are concerned with, (1) discovering the disease in any locality; (2) searching out the human sources of infection; (3) ascertaining the particular species of mosquito which is transmitting the disease, and learning the finer points in its life history; (4) studying the circumstances which make a population susceptible; (5) correlating the results to give a clear picture of the distribution of the disease and the manner in which it has arisen; (6) on this finding, to formulate a precise plan of preventive action.

### Historical.

On August 21, 1917, at an Epsom hospital benign tertian malarial parasites were found in the blood of a soldier who had never been out of England, and whose history suggested that he contracted the disease in camp near Sheerness. Then followed the famous investigation at the Kentish port, and in a very short time a number of similar home-infected military cases were discovered. As investigation extended to the local civil population the same thing was discovered, and, besides certain definite foci of infection, a seasoned incidence, with its maximum in September, was also proved. Another point of even greater importance was elucidated, namely, that at least one of the cases had originated before any troops whatever had returned from the East, and on these findings, together with the local knowledge that as late as 1870 " nearly everyone in Queenborough suffered from ague," it was concluded that among the civilian cases there were some definite examples of true indigenous disease which had never quite disappeared from the locality. At this time no difficulty was found in catching a number of adult *Anopheles maculipennis* in the district, and their larvæ were abundant in ponds, ditches, and flooded areas near the outskirts of the town; that these insects differed in any essential respect from their sub-tropical brothers was amply disproved by deliberately feeding them on malarial patients and proving that they were perfectly efficient hosts for the benign tertian parasite. A full account of this work was published in the journal of the R.A.M.C., November 1917, p. 615, and it is unnecessary here to detail the relatively obvious methods which were taken to counteract the menace locally. But with this example so strikingly proved it is not surprising that similar investigations were carried out as rapidly as possible in other districts. To the wider distribution thus determined we shall return in a later paragraph, and for the moment confine our attention to the house-to-house incidence.

### Malarial Houses.

It is extremely important to note, however, that cases were not scattered at random throughout the town, but were definitely localised to certain streets and houses. So noticeable was this that the findings provided some striking examples of " malarious houses," which were aptly compared with the " yellow fever houses " that so long puzzled epidemiologists before this fever was known to be carried by the domestic or *stegomyia* mosquitoes. In one instance a landlady proved to be an apparently healthy carrier, and indirectly infected at intervals a number of lodgers at her house. Such transference is not in itself surprising, but combined with the distributional evidence it brings us to realisation that the " localised character of distribution of cases of malaria in England, and particularly the incidence in houses and families, indicates that the mosquito concerned (*A. maculipennis*) has the habit of remaining most of its life within or in the immediate vicinity of the house where it obtained its first meals of blood." Herein lies the first great difference to the corresponding tropical situation where the insects travel far and wide, and for this we would suggest a climatic explanation. But the lesson which it carries for the health worker is that there are a set of conditions likely to arise in poorer-class dwellings which constitutes them " dangerous." It is beyond the scope of this note to describe them in more detail than low-pitched, dark houses, having plentiful day-time resting corners for the insects. Colonel James has entered very fully into the subject, and the point we would emphasise is that the health worker has to be on the look-out for mosquito-favouring circumstances in such dwellings, and where possible examples of family infection have occurred they must essentially search for all such " malarious houses," and, apart from preventing undue entry of troops or strangers, do all in their power to bring about local improvement.

### The Malarial Map.

If we look at a map of England charted to show the past and present distribution of the disease, there are a number of interesting points to be made

**The Malarial Survey in England**—(*continued*).

out. The distribution of "ague" in the nineteenth century seems to have been mainly along the East Coast and in the Eastern and South-Eastern Counties. A certain amount occurred in Hampshire, opposite the Isle of Wight, and isolated areas near some of the older ports on the West are also marked in. But when this incidence is compared with the findings of the most recent survey (1917-1918), it is noticed that although cases of apparently indigenous malaria occurred over similarly distributed but more widely separated localities, yet in only one county (Kent) was there any considerable local spread of the disease. This circumstance is difficult to explain on grounds other than the fact that around the mouth of the Thames and the partially reclaimed marshes was found the last *real* stronghold of the original English ague, but it can be claimed that in this district definite proof has been obtained that there do remain in England a few areas where true indigenous English malaria persists.

Sources of Actual Infection.

Thus we must conclude that there are both insects capable of carrying the disease and circumstances in which only a century ago they quite successfully did so. Hence the obvious danger of the malarial carrier in this country and the need for his earliest possible detection. With regard to cases returning to the country from the East, although each relapse of malaria in these men can be quickly cured by quinine, no present treatment suffices to effect a permanent cure in a reasonably short period of time. From the map showing the present distribution of the returned Ministry of Pensions cases, it is at once realised that these men are now scattered broadcast throughout the country. Notification since the Ministry of Health's regulations of March 1, 1919, has done much to keep accurate the official knowledge of the whereabouts of these possible carriers, but we know the limitations of such circumstances in practice, and it is for this reason that we would call the attention of the humblest health worker to the absolute necessity that he or she should acquire a reasonably practical knowledge of malaria as a disease, and should bear in mind that it is at the moment of as great importance to the health returns of the future as many of those classical conditions which time and custom have made the special concern of the public health services in England.

